# Increased risk factors associated with lower BMD in antiretroviral-therapy–naïve HIV-infected adult male

**DOI:** 10.1186/s12879-021-06263-9

**Published:** 2021-06-09

**Authors:** Patricia Atencio, Alfonso Cabello, Francisco M. Conesa-Buendía, Ramón Pérez-Tanoira, Laura Prieto-Pérez, Irene Carrillo, Beatriz Álvarez, Rosa Arboiro-Pinel, Manuel Díaz-Curiel, Gabriel Herrero-Beaumont, Aránzazu Mediero, Miguel Górgolas

**Affiliations:** 1 Infectious Diseases Division, Fundación Jiménez Díaz University Hospital, Universidad Autónoma de Madrid, 28040 Madrid, Spain; 2 Bone and Joint Research Unit, IIS-Fundación Jiménez Díaz, Universidad Autónoma de Madrid, 28040 Madrid, Spain; 3 Internal Medicine, Metabolic Bone Diseases Unit, Fundación Jiménez Díaz University Hospital, Universidad Autónoma de Madrid, 28040 Madrid, Spain

**Keywords:** HIV, Bone comorbidity, Naïve patients, DXA, BMD

## Abstract

**Background:**

Low BMD (bone mineral density) has been described as a non–AIDS (Acquired Immune Deficiency Syndrome)-related event in HIV (human immunodeficiency virus)-patients but it is poorly studied in young HIV-infected men who have received no previous antiretroviral therapy.

**Methods:**

A cross-sectional study of 245 naïve-HIV-infected men over 21 and under 50 years old who voluntary attended the Infectious Disease Division appointment in Hospital Fundación Jimenez Díaz in Madrid, from January 1st, 2014 to September 30th, 2017. All subjects underwent a baseline DXA scan (dual energy x-ray absorptiometry) performed prior to start antiretroviral treatment. Further, all patients who started treatment between May 1st and September 30th, 2017 were invited to participate in a substudy on bone mineral metabolism. All the information was collected through clinical history and complementary questionnaire.

**Results:**

The mean age was 36.4 years, been 68% Caucasian, 29.3% Latin American and 2.7% African race. At the time of diagnosis, 91% of patients had stage-A (median CD4+ T-cell 481cells/μL, IQR, 320–659). 10% had a count below 200 CD4 cells/μL, and 40% had a CD4/CD8 cell-count-ratio below 0.4. Regarding lifestyle and risk factors, 14.1% presented underweight, 36.1% were not engage in any regular exercise, 51.9% were active smokers and 35.3% reported drug use. Low levels of vitamin D were seen in 87.6% of the study participants. Low BMD (Z-score <- 2.0) was found in 22.8% of the patients. It was only observed a significant association of Z-score in lumbar spine (LS) with CD8 and the CD4/CD8 ratio, and with alcohol for femoral neck (FN) measurement.

**Conclusions:**

We find prevalence of increased bone involvement among naïve HIV-infected men under 50 years old. Further studies are necessary to evaluate if changes in actual guidelines are needed to assess BMD measurements in HIV-infected adult male patients under 50.

## Background

With the development of antiretroviral therapy (ART), people living with HIV (PLWH) have considerably improved life expectancy [1]. In recent years, however, there has been a steady rise in so-called non–AIDS (Acquired Immune Deficiency Syndrome)-related events, such as cardiovascular events, non–AIDS-defining tumors, as well as bone involvement and abnormally low bone mineral density (BMD) [2].

Bone involvement, defined as the presence of osteopenia or osteoporosis on a dual energy x-ray absorptiometry (DXA) scan [3, 4], has been the subject of numerous studies, and have focused on bone toxicity associated with ART. A number of clinical trials have described reduced BMD during the first or second year of ART, independently of the type of therapy [5, 6], in particular, tenofovir disoproxil fumarate (TDF) and protease inhibitors (PIs) have been associated with higher rates of medium- and long-term bone toxicity [7]. As a result, the primary international practice guidelines recommend performing a DXA scan in HIV-infected individuals over the age of 50 years [1, 7].

The factors that contribute to increase bone involvement are widely known and include age, vitamin D deficiency, tobacco and alcohol consumption, a sedentary lifestyle, among others [8, 9]. In addition to these factors, PLWH exhibit a marked proinflammatory state even after ART start [10]. Our purpose is to provide information on bone situation in antiretroviral-therapy–naïve HIV-infected adult male under 50 years of age. It is expected that study participants will have the presence of an increased bone involvement than similar uninfected population, even prior to initiation of ART and under 50 years [11, 12], which could suggest the beginning of the bone study prior to 50 years of age.

The aim of this study is to assess bone involvement and risk factors that may contribute to the onset of low BMD among adult HIV-infected men (under 50 years of age), who are naïve to ART.

## Methods

A cross-sectional study of HIV-infected men over 21 and under the age of 50 years who were naïve to ART in a tertiary teaching hospital in central Madrid, Spain.

### Subjects and study design; inclusion criteria and ethical concerns

Two hundred forty-five adult men (over 21 and under the age of 50 years) who voluntary attended the Infectious Disease Division appointment in Hospital Fundación Jimenez Díaz in Madrid, a tertiary hospital in the center of Madrid, from January 1st, 2014 to September 30th, 2017, with a recent diagnosis of HIV infection, without previous HIV treatment. All subjects underwent a baseline DXA scan (HOLOGIC QDR 4500C, Marlborough, MA, USA) performed prior to start ART. Further, all patients who started treatment between May 1st and September 30th, 2017 were invited to participate in a substudy on bone mineral metabolism. The hospital attends more than 3000 people living with HIV (PLWH), over 95% of newly infected patients are men who have sex with men (MSM). The protocol for this study was approved by the clinical research ethics committee of the Hospital Fundación Jimenez Diaz (approval code: PIC, 155–2016, approved on 20 December 2016) and is in adherence with the tenets of the Declaration of Helsinki. All patients provided signed informed consent before being included in the study.

Exclusion criteria were as follow: patients over 50 years old, previous HIV or bone-targeting treatment (denosumab, vitamin D), treatment with systemic corticosteroids in case the patient was taking corticosteroids or had taken them for more than 3 months at a dose equivalent to 5 mg of prednisolone per day or more, diagnosed of diabetes, rheumatologic and renal diseases, thyrotoxicosis, advanced liver disease, malabsorption syndrome, neoplasms or previous fragility fractures (pathological fracture produce with a minimal trauma such as falls from a standing height and blows).

All new diagnose, naïve HIV infected individuals that came to the clinic without any of these criteria were attended to be included in the study.

### Measurements and reference values

We gathered such epidemiologic data as age, race, and country of birth. Lifestyle-related aspects used as study variables included alcohol, tobacco, and drug consumption, physical activity, and approximate calcium intake. Additionally, anthropometric data were collected for all patients. Blood test was done fasting, measured by Advia 2400 system (Siemens®, Munich, Germany) for values related to calcium, phosphorus, and vitamin D. Immunological and virologic parameters (i.e., CD4+, CD8+, and HIV-1 viral load) were measured by PCR (Roche, Basel, Switzerland).

Underweight was defined as a body mass index (BMI) of < 20 kg/m^2^ [13, 14]. The values related to bone and mineral metabolism provided by the hospital laboratory were as follows: 25OH Vitamin D (30–50 ng/ml), and parathormone (PTH) (10–70 pg/ml) [15, 16]. Patients considered smokers if they were current or past tobacco users; consumers of alcohol if their total intake was over 30 g/day; and drug users [cocaine, mephedrone, amphetamines, ketamine, gamma hydroxybutyrate (GHB)] if any of these drugs were taken at least once weekly. For the purposes of this study, sufficient physical exercise was a minimum of 120 min per week. Three servings of calcium-rich foods (e.g., milk, cheese, other dairy products) daily was considered an appropriate intake [17].

DXA scan was performed before the start of ART. BMD was determined by bone densitometry in lumbar spine (LS), and femoral neck (FN). Being our sample subjects under 50 year and according to the World Health Organization (WHO) guidelines, the subjects were classified with Z-score, considering low BMD values under − 2.0 [4].

### Statistical analysis

Qualitative variables were expressed in terms of frequency and percentages. Based on the results of the Kolmogorov-Smirnov test for normality, quantitative variables were measured as either mean and standard deviation or median and interquartile range (IQR). Qualitative variables were analyzed using the Chi-squared test or Fisher’s exact test. Quantitative variables were compared using Student’s t test. For all determinations, we used R software version 3.6.0 (R Core team (2020); R Foundation for Statistical Computing, Vienna, Austria), and statistical significance was set at *p* < 0.05.

## Results

### Epidemiology and lifestyle

A total of 245 patients were included, all of whom were men. The main patient characteristics appear in Table 1. The median age of the patients was 36.4 years, 68% of whom were Caucasian (87.7% Spanish), and 29.3% Latin American. All patients had been infected with the HIV virus through sexual intercourse (MSM). At the time of diagnosis, 91% of patients had stage-A disease according to the classic CDC (Center for Disease Control) classification system, and the median CD4+ T-cell count was 481 cells/μL (IQR, 320–659). Ten percent of these stage-A patients had a count below 200 CD4 cells/μL, and 41% had a CD4/CD8 cell-count ratio below 0.4. (Table 1). Among evaluated patients, 32.62% had high viral load (> 100,000 copies/ml) (Table 1).

**Table 1:** Main characteristics of patients (p)

***BASELINE CHARACTERISTICS***	***N = 245 PATIENTS***
**Sex**	
*Male*	100%
*Female*	0%
**Route of transmission MSM**	100%
**Age (mean)**	36.4(IQR: 30–40)
**Race**	
*Caucasian*	68%
*Latin American*	29.3%
*African*	2.7%
**Viral Stage**	
*A*	91%
*B*	6%
*C*	3%
**High viral load (> 100,000 copies/ml)**	32.62%
**CD4 (median)**	481 cells/μL (IQR: 320–659)
**CD4 < 200 cells/**μL	10%
**CD4 / CD8 ratio < 0.4**	41%
**Coinfection (HCV antibody/**	1.22%
**Coinfection HVB antigen S**	0.4%
**Time between HIV diagnosis and DXA scan (median)**	3.3 months
**Z-score**	Normal (> − 2): 77.2% (189 p) Low BMD (<−2.0): 22.8% (56 p)

**Fig. 1:**
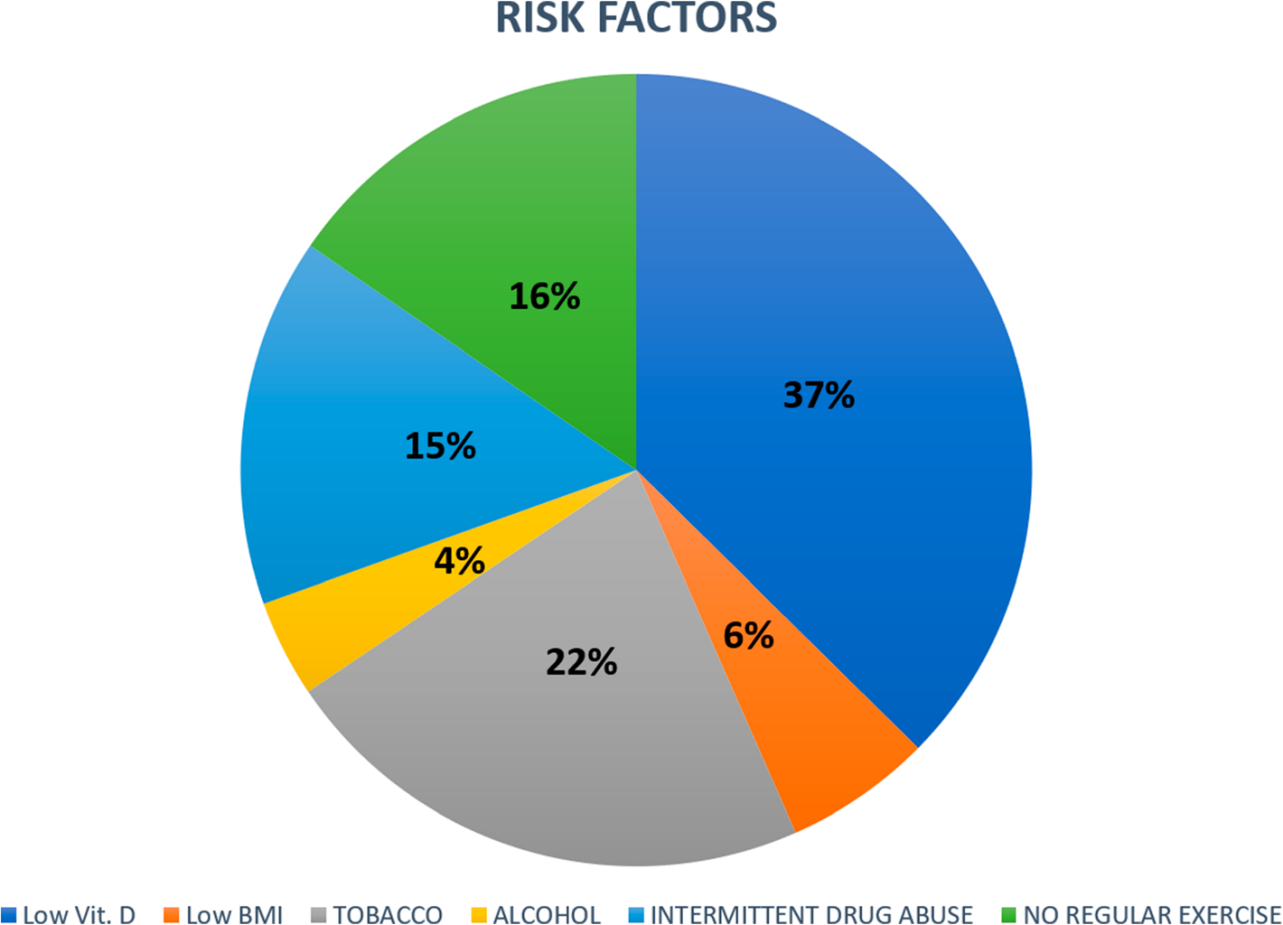
Risk factors associated with bone comorbidity in HIV patients. The figure represents the percentage of incidence of each of the risk factors analyzed in the study population

Regarding lifestyle patterns and risk factors associated with lower BMD, 14.1% presented underweight with low BMI, 36.1% were not engage in any regular exercise, 51.9% were active smokers, 35.3% reported drug use, and 9.3% drank alcohol habitually. Abnormally low levels of vitamin D were seen in 87.6% of the study participants. Based on these values, (Fig. 1) represents the percentage of incidence of each of the risk factors analyzed in the study population.

### Bone alterations in naïve patients

Before starting ART after HIV diagnosis, patients underwent DXA in order to see the initial bone state and be able to compare it with the final stage after ART. The median time elapsed between diagnosis and DXA was 3.3 months (Table 1). As all the patients were less than 50 years old, the Z-score was calculated, finding that 22.8% of them had a Z-score below − 2.0 (Table 1). No significant differences were observed for the other risk factors studied in these patients.

In order to understand if any of the studied parameters affect Z-score at any location (LS or FN), a linear regression study was performed (Tables 2 and 3): We only observed a significant association of Z-Score in LS with CD8 and the CD4/CD8 ratio (Table 2) and also with alcohol for FN measurement (Table 3).

**Table 2:** Comparative analysis of linear correlation between Z-Score in LS and baseline parameters studied in HIV patients. The following table summarizes the results of these models using the coefficient (coef.), their 95% confidence interval (95% CI), and the *p*-value. *P* < 0.05 = significant

***Variable (***vs ***Z-score CL)***	***Coef.***	***(95% CI)***	***P***
Age	−0.002	(−0.018, 0.015)	0.847
BMI	0.000	(−0.001, 0.000)	0.546
Ca	−0.110	(−0.463, 0.243)	0.539
P	−0.049	(−0.240, 0.142)	0.613
Vit. D	0.012	(−0.013, 0.036)	0.344
Albumin	−0.283	(−0.770, 0.205)	0.254
CD4	−0.012	(−0.063, 0.039)	0.644
CD8	0.030	(0.004, 0.055)	0.022
CD4/CD8 ratio	−0.588	(−1.092, − 0.084)	0.022
Viral load	0.000	(−0.131, 0.131)	0.997
**Tobacco**			
Yes	0.140	(−0.179, 0.460)	0.387
**Alcohol**			
Yes	0.016	(−0.462, 0.495)	0.946
**Drugs**			
Yes	0.092	(−0.269, 0.452)	0.617
**HIV Stage**			
B	0.145	(−0.459, 0.749)	0.636
C	0.017	(−0.825, 0.858)	0.969
**Dairy**			
No	0.258	(−0.644, 1.161)	0.571
**CD4**			
< 200	0.173	(−0.282, 0.629)	0.454
**CD4/CD8 ratio**			
< 0.4	0.368	(0.084, 0.653)	0.011
**Viral Load**			
High	0.223	(−0.168, 0.613)	0.262

**Table 3:** Comparative analysis of linear correlation between Z-Score in FN and baseline parameters studied in HIV patients. The following table summarizes the results of these models using the coefficient (coef.), their 95% confidence interval (95% CI), and the *p*-value. *P* < 0.05 = significant

***Variable (***vs ***Z-score*** CF***)***	***Coef.***	***(95% CI)***	***P***
Age	0.013	(−0.001, 0.026)	0.061
BMI	0.000	(−0.000, 0.001)	0.207
Ca	−0.098	(−0.394, 0.198)	0.515
P	−0.074	(−0.234, 0.087)	0.366
Vit. D	0.013	(−0.004, 0.030)	0.124
Albumin	0.110	(−0.301, 0.521)	0.599
CD4	−0.015	(−0.057, 0.028)	0.497
CD8	0.013	(−0.009, 0.034)	0.239
CD4/CD8 ratio	−0.385	(−0.808, 0.038)	0.074
Viral Load	−0.049	(−0.162, 0.065)	0.397
**Tobacco**			
Yes	0.249	(−0.020, 0.518)	0.069
**Alcohol**			
Yes	−0.561	(−0.957, − 0.165)	0.006
**Drugs**			
Yes	−0.006	(−0.327, 0.315)	0.970
**HIV Stage**			
B	0.134	(−0.377, 0.644)	0.606
C	0.033	(−0.653, 0.719)	0.924
**Dairy**			
No	0.529	(−0.026, 1.084)	0.062
**CD4**			
< 200	0.252	(−0.139, 0.644)	0.205
**CD4/CD8 ratio**			
< 0.4	0.198	(−0.044, 0.440)	0.108
**Viral load**			
High	0.073	(−0.289, 0.434)	0.692

## Discussion

HIV-infected people are at risk of developing increased bone fragility caused by a loss of BMD and consequently increased risk of bone fractures. The cause of bone loss in HIV is multifactorial, including traditional risk factors, ART, HIV viral proteins, and chronic inflammation triggered in these patients by the virus [18]. In this article we detail the data obtained from this preliminary study with HIV patients in adulthood, in the initial stages of ART and in ages that do not reach old age and consequent bone fragility.

How it can be extracted from the data obtained in our work, the results reveal a significant prevalence of bone involvement among newly diagnosed HIV-infected men before initiation of ART without any known secondary causes of osteoporosis, with 22.8% of the patients with a BMD lower than that expected for their age (Z-score < − 2.0). Additionally, a high percentage of these patients have low levels of vitamin D (87.6%).

Our data are similar to what has been previously described in other studies with patients of similar age groups. In this sense, Paccou J et al. [12] involving 49 naïve men, mean age was 31.6 (±7.7) years demonstrated that the prevalence of low BMD was 24.5% [95% CI, 13.3–38.9], similar to our findings. In another study by Ceballos et al. [11] involving 70 naïve men, mean age 31 years (19–50), Low BMD (Z-score < − 2.0) was found in 13% of the patients.

It is highly likely that the lifestyle of the study population, is an important factor behind such high rates of bone involvement. In our findings we observed tobacco use (51.9%), no regular exercise (36.1%) and intermittent drug abuse (35.3%) as the most prevalent risk factors in our cohort. Previous studies have described that these factors may contribute to a decrease in BMD and an increased risk of fracture among patients infected with HIV. Nearly all these conditions have been found to be more prevalent among PLWH and ART experienced [19–22], and several cohort studies have shown increased rates of bone fracture among HIV-infected patients compared to uninfected population [23, 24].

Special attention should be given to vitamin D status and its impact on bone metabolism in these patients. In our cohort, 87.6% of the patients had low levels of vitamin D. In recent years, a number of studies have suggested that patients living with HIV infection have a high prevalence of vitamin D deficit independently of their geographic origin [25–27], similar to findings in adults generally (HIV 70.3% compared with 79.1% of HIV-negative adults) [28]. In addition to its deleterious impact on patients with HIV infection, vitamin D deficiency is a well-established risk factor for bone disease within the general population [29]. Indeed, recent publications suggest that the functions of vitamin D go beyond the skeleton, and that vitamin D may play a role in regulating cardiovascular and immunologic parameters [30–32]. Though some studies have described a protective role played by vitamin D in which this vitamin prevents loss of bone mass [33], much remains unknown as to the degree to which vitamin D deficiency contributes to this loss and to an increase in risk of fracture among HIV-infected patients, so vitamin D should be included in the screening of bone fragility in this population [34].

Currently, the primary guidelines and international consensus statements recommend that patients who are infected with the HIV virus undergo bone testing if they are over the age of 50 years [7, 35, 36] or with a history of pathologic fractures. These publications further advise clinicians to avoid ART regimens that pose a risk of bone toxicity, such as tenofovir disoproxil fumarate (TDF) and protease inhibitors, if the patient has existing bone involvement or fragility fracture [1, 7, 22, 37]. Our findings suggest that this recommendation may be revised, as over 22% of our study population, which consisted of MSM under age 50, had low BMD levels for their age. As there is still no curative treatment for HIV infection, it is foreseeable that these patients will continue requiring ART for years to come, thus putting them at an increased risk of loss of bone mass. Likewise, when doing a logistic regression study of Z-score, assuming in this case normal and non-normal values, versus the same parameters under study; only the CD4/CD8 ratio appears to be associated in this case (Table 4). Therefore, this data leads us to think about the possible relationship between viral load and infection itself with the progressive bone deterioration of the HIV patient.

**Table 4:** Comparative analysis of logistic correlation between Z-Score (qualitative binary variable: normal range or non-normal range) and baseline parameters studied in HIV patients. The following table summarizes the results of these models using the odds ratio (OR), their 95% confidence interval (95% CI), and the *p*-value. *P* < 0.05 = significant

***Variable***	***OR***	***(95% CI)***	***P***
Age	1.01	(0.97, 1.04)	0.764
BMI	1.00	(1.00, 1.00)	0.691
Ca	1.25	(0.58, 2.72)	0.578
P	1.26	(0.84, 1.91)	0.246
Vit. D	0.98	(0.93, 1.03)	0.345
Albumin	0.86	(0.30, 2.50)	0.776
CD4	1.01	(0.91, 1.13)	0.800
CD8	0.95	(0.89, 1.01)	0.123
CD4/CD8 ratio	3.41	(1.19, 9.9)	0.022
Viral Load	1.05	(0.74, 1.37)	0.726
**Tobacco**			
Yes	0.62	(0.30, 1.28)	0.204
**Alcohol**			
Yes	2.04	(0.66, 8.95)	0.265
**Drugs**			
Yes	0.71	(0.28, 1.65)	0.447
**HIV Stage**			
B	0.27	(0.01, 1.44)	0.218
C	0.55	(0.03, 3.30)	0.580
**Dairy**			
No	1.25	(0.17, 6.44)	0.801
**CD4**			
< 200	0.92	(0.29, 2.44)	0.876
**CD4/CD8 ratio**			
< 0.4	0.52	(0.26, 0.99)	0.050
**Viral Load**			
High	0.60	(0.23, 1.41)	0.256

Among the limitations of the study, it should be noted that this is a single-center study may have influenced the interpretation of some of our results, as a similar study performed in another geographic location may find an increase or a decrease in the same parameters observed, mostly due differences in demographic, social and lifestyle. A substudy by Carr [38], with a total of 424 ART-naïve participants in six continents, with a mean age of 34 (10.1) years, showed that 1.9% of patients had osteoporosis and 35.1% had low BMD;. A second limitation concerns the lack of a study group consisting of individuals not infected with the HIV virus; nonetheless, our results can be contrasted with well-established findings from studies conducted in the general population. Though our cohort consists of individuals who have lived with HIV infection for a short time, certain bias may have been introduced in this regard, as some of the patients studied had no previous tests with a negative result, thus making exact data on cumulative time of infection unavailable. However, the fact that over 90% of patients had stage-A disease indicates an appropriate degree of homogeneity. The most significant limitation of our research stems from the lack of a validated means of screening for bone involvement in patients with HIV infection. Indeed, neither the Fracture Risk Assessment Tool (FRAX) nor densitometric methods have been validated in young people or in the population of individuals living with HIV infection [1, 35, 39, 40]. Despite the limitations discussed above, we believe that the large number of patients included in this study lends validity to its findings and makes the case for baseline examinations of bone density to improve the clinical management of young men living with HIV infection.

In short, the inclusion of DXA densitometry measurements and bone marker analysis as part of baseline evaluation of HIV-infected patients, although alone they would not be used to diagnose the disease in particular but would provide clinical data to the physician to improve the health of the patient’s bone mass, improve lifestyle habits that promote this bone comorbidity and avoid prescribing antiretroviral therapy that leads to bone loss. Similarly, a diagnosis of low BMD at an early age would affect the follow-up approach given to certain patients who by age do not undergo or consider densitometric parameters in the same way. As noted above, an increased risk of bone fracture has been found among patients with HIV infection compared to HIV-negative people; however, these studies have not found a correlation between abnormal DXA measurements and subsequent fracture [40]. As a result, we argue that the most appropriate strategy in treating young patients with bone involvement evidenced in DXA scanning should be in accordance with the general recommendations of scientific societies and should seek to control the risk factors associated with both antiretroviral and therapeutic treatment for bone comorbidity from the outset of follow-up.

## Conclusions

We find a significative prevalence of bone involvement among naïve HIV-infected men under 50 years old. Further studies are necessary to evaluate if BMD assessment should be recommended in HIV-infected patients under 50 years of age.

## Data Availability

The datasets used and/or analysed during the current study are available from the corresponding author on reasonable request. All data generated or analyzed during this study are included in this published article.

## Abbreviations

AIDS: Acquired Immune Deficiency Syndrome
ART: Antiretroviral therapy
BMD: Bone mineral density
BMI: Body mass index
CDC: Center for Disease Control
DXA: Dual energy x-ray absorptiometry
FN: Femoral neck
FRAX: Fracture Risk Assessment Tool
GHB: Gamma hydroxybutyrate
HIV: Human immunodeficiency virus
IQR: Interquartile range
LS: Lumbar spine
MSM: Men who have sex with men
PCR: Polymerase Chain Reaction
PIs: Protease inhibitors
PLWH: People living with HIV
PTH: Parathormone
TDF: Tenofovir disoproxil fumarate
WHO: World Health Organization
